# Management of Neglected Pubic Symphysis Diastasis and Sacroiliac Joint Disruption With Erectile Dysfunction in Polytrauma: A Case Report

**DOI:** 10.7759/cureus.52958

**Published:** 2024-01-25

**Authors:** Dilip Kumar Naidu, Aswin S, Madhan R, Gowthaman N, Kevin Dhas

**Affiliations:** 1 Orthopaedics, SRM Medical College Hospital and Research Centre, Chengalpattu, IND; 2 Orthopaedic Surgery, SRM Medical College Hospital and Research Centre, Chengalpattu, IND

**Keywords:** road traffic injuries, sacroiliac joint fixation, sacroiliac screw, pelvic ring injury, erectile dysfunction, polytrauma, post traumatic erectile dysfunction, neglected orthopaedic injury, neglected pubic diastasis injury, pelvic fracture

## Abstract

Pelvic fractures are the most common among patients sustaining high-energy trauma. They are associated with high morbidity and mortality rates, often because of high blood loss and injury to the lumbosacral plexus, genitourinary system, and gastrointestinal system. The age, complexity of the pelvic fracture, and pubic symphysis diastasis would represent risk factors for erectile dysfunction after major and neglected pelvic injuries; the neglected pelvic ring injuries could cause disabilities that manifest with symptoms like pain, lower limb length discrepancy, standing or sitting imbalance and even sexual dysfunction. Herein, we report a case of a young adult who sustained polytrauma and was diagnosed with neglected pubic symphysis diastasis, sacroiliac joint disruption, and erectile dysfunction. The patient regained erectile function after the surgical management of pubic symphysis diastasis and sacroiliac joint disruption.

## Introduction

Openbook fractures result from high-energy anteroposterior forces applied to the pelvic ring [1]. Although rare, low-energy trauma can also cause open-book-type injuries. Failure to properly treat such injuries can result in long-term complications, including chronic pain, gait disturbances, sitting discomfort, neurological deficits, and urogenital problems [2]. Erectile dysfunction is a common and distressing consequence of pelvic ring fractures, particularly in younger patients, but is often overlooked in patients with polytrauma and potentially life-threatening injuries [3]. Inadequate management of unstable pelvic fractures can result in significant long-term morbidities. Pain is the most common complaint among patients with late malunion or non-union of the pelvic ring [4-6]. This case report presents the successful surgical management of neglected pubic symphysis diastasis and sacroiliac joint disruption, resulting in the improvement of erectile dysfunction symptoms.

## Case presentation

**Figure 1:**
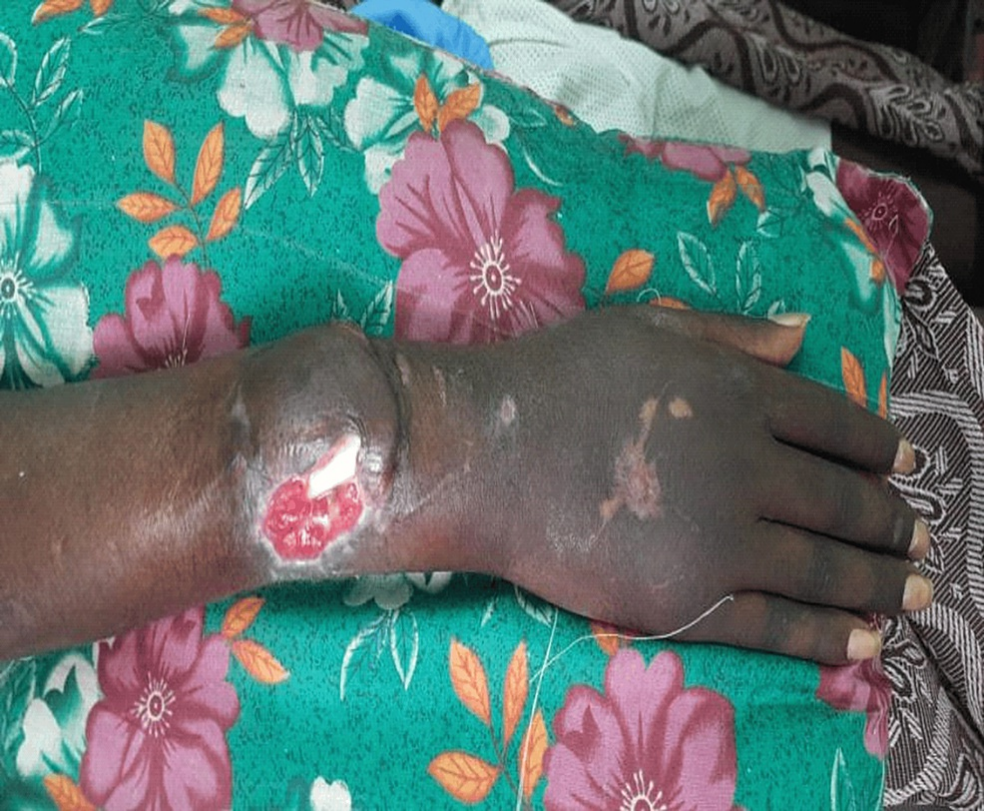
Wound in the posterolateral aspect of the distal right forearm with right distal ulna bone exposed

**Figure 2:**
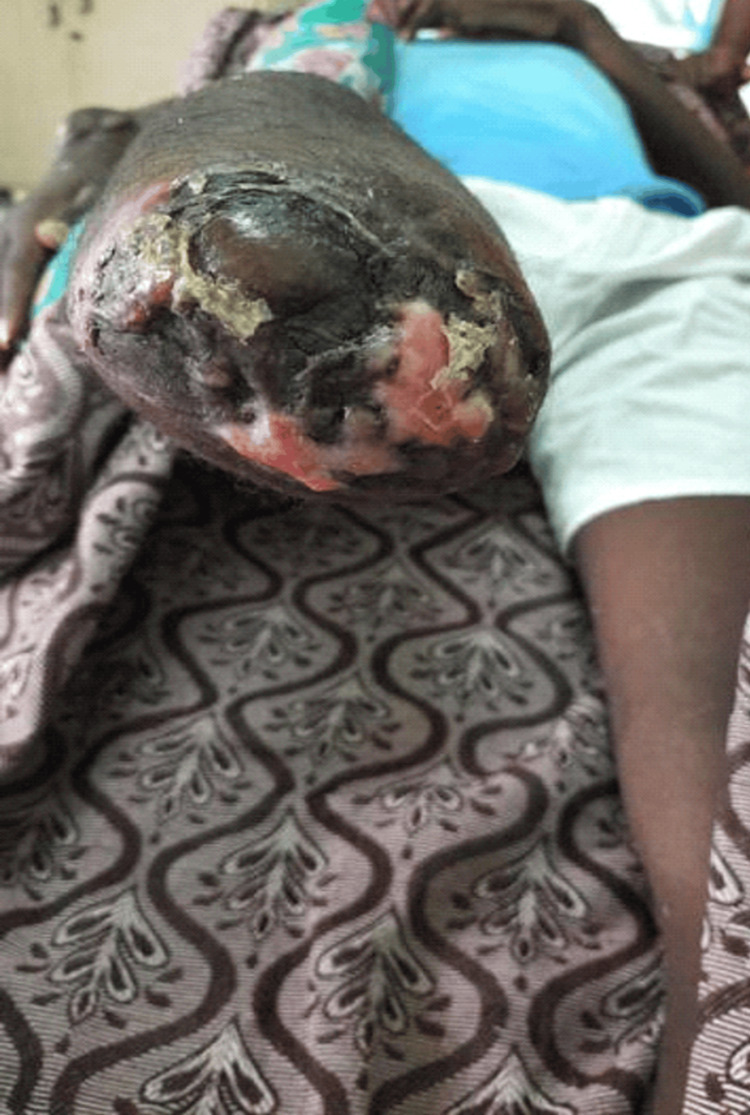
Non-healing ulcer over right below-knee stump

A 23-year-old male with no known comorbidities presented with complaints of inability to sit, or turn around in bed, wound of size 3x2 cms with active discharge with exposed underlying bone in the posterolateral aspect of the distal right forearm, and erectile dysfunction. The patient had an alleged history of a road traffic accident with a 2-wheeler (the patient was the rider) directly hitting a 4-wheeler. He sustained an injury to his hip, right leg, and right forearm. The patient was initially admitted to a nearby hospital and was diagnosed with a crush injury on the right leg and comminuted distal, both bone fractures of the right forearm, and a series of procedures were performed. As procedure 1, right below-knee guillotine amputation and below the elbow slab were applied on the right forearm. After 2 weeks, the below-knee amputation stump was infected; so, the patient underwent procedure 2, a wound debridement of the right below-knee amputation stump and K-wire fixation for both bone fractures of the right forearm. After 3 weeks he underwent procedure 3, a split skin graft for the right below-knee stump amputation raw area, and a peri-umbilical perforation-based abdominal flap for the raw area of the right forearm. Then, after 3 weeks right forearm K-wire implant was infected and the patient underwent procedure 4, where K-wire removal from the right forearm was performed. At the time of presentation, local examination of the right forearm had a raw area of size 3 × 2 cm present in the posterolateral aspect of the distal forearm adjacent to the flap, distal ulna bone exposed (Figure 1), wrist range of movements absent, flickering movements of fingers present, radial artery pulse present, and sensation decreased in all dermatomes of the right hand. A nonhealing ulcer was present over the below knee stump right (Figure 2). On hip examination, tenderness was present over the pubic symphysis and right sacroiliac joint, and there was pain and a reduced range of movement in the right hip.

**Figure 3:**
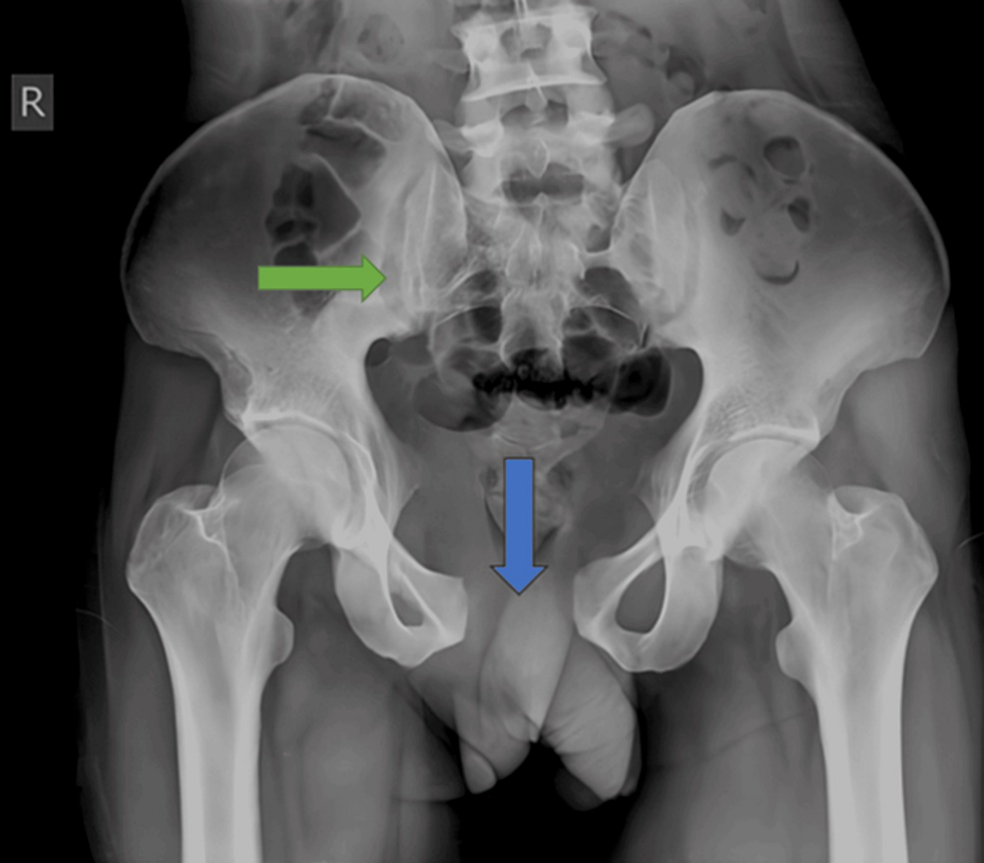
X-ray pelvis anteroposterior view showing pubic symphysis diastasis (marked with blue arrow) and sacroiliac joint disruption right (marked with green arrow)

**Figure 4:**
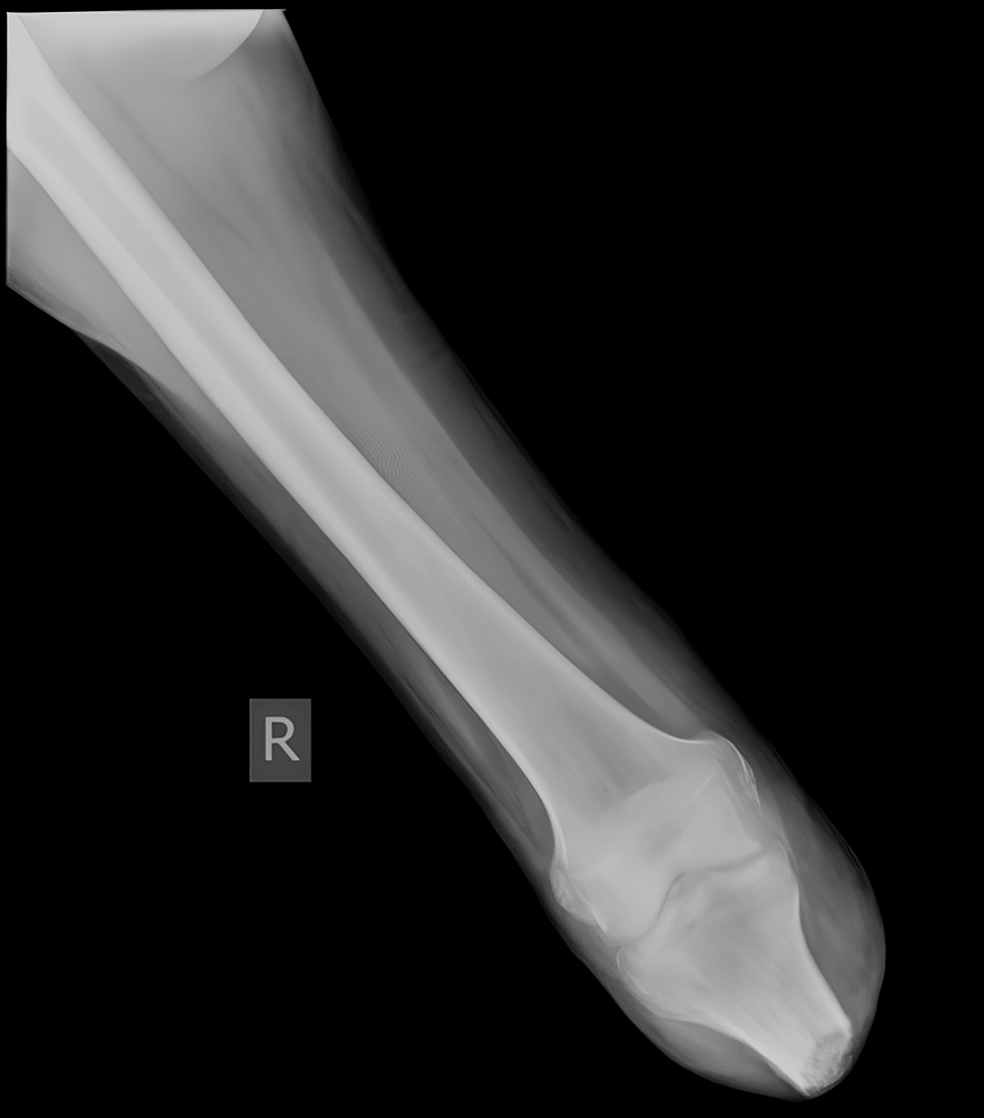
X-ray anteroposterior view showing below-knee amputation stump right

**Figure 5:**
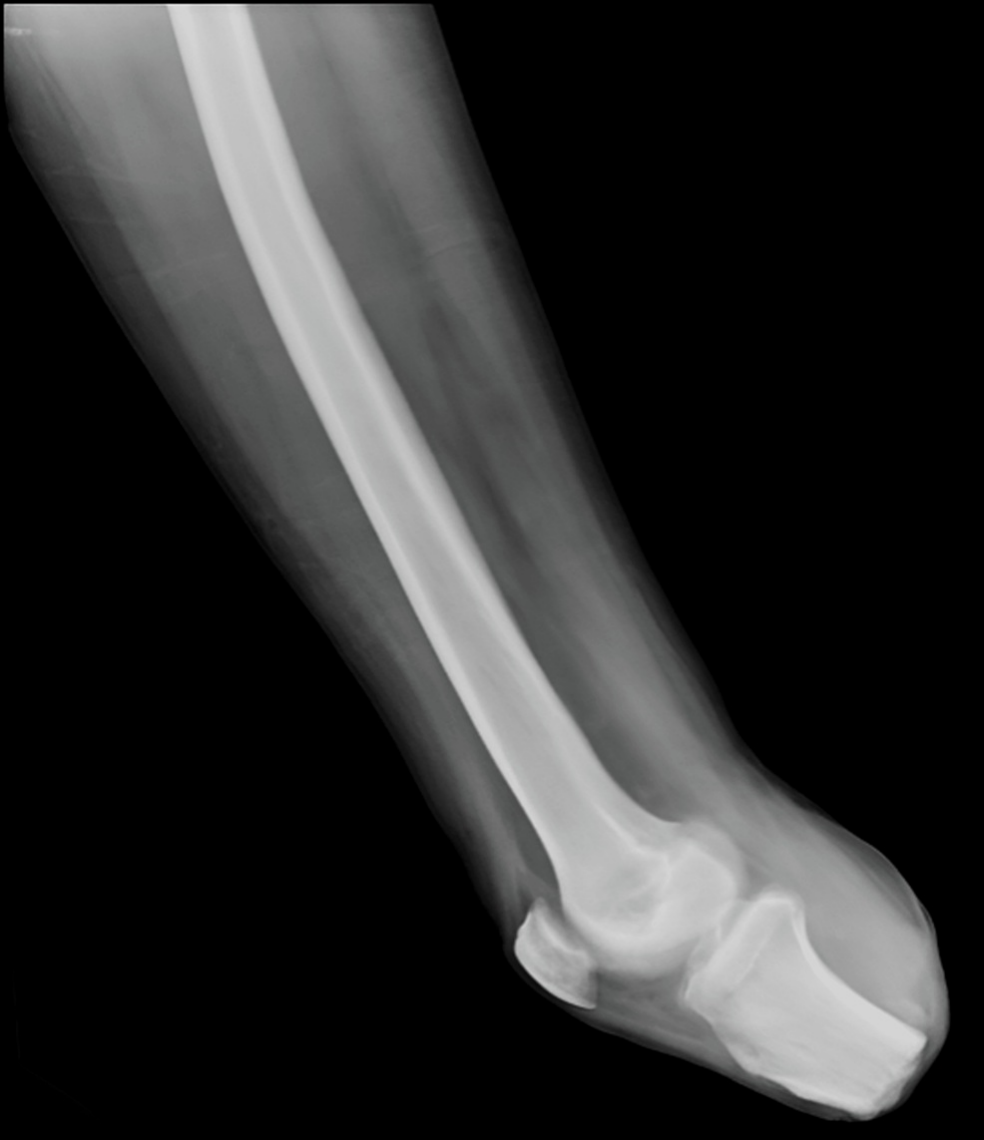
X-ray lateral view showing below-knee amputation stump right

**Figure 6:**
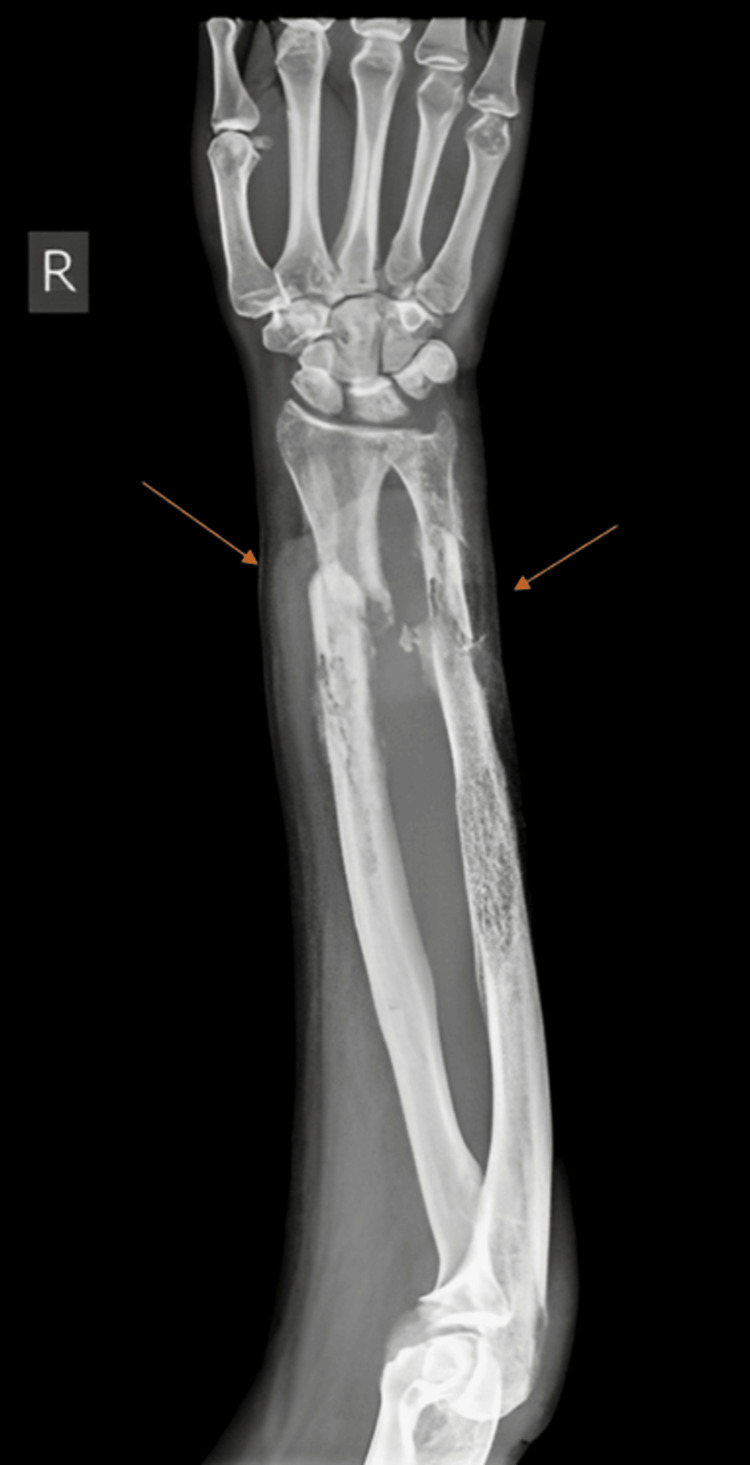
X-ray of the right forearm anteroposterior view showing comminuted fracture with acute osteomyelitis of both bone distal forearm right (marked with orange arrow)

**Figure 7:**
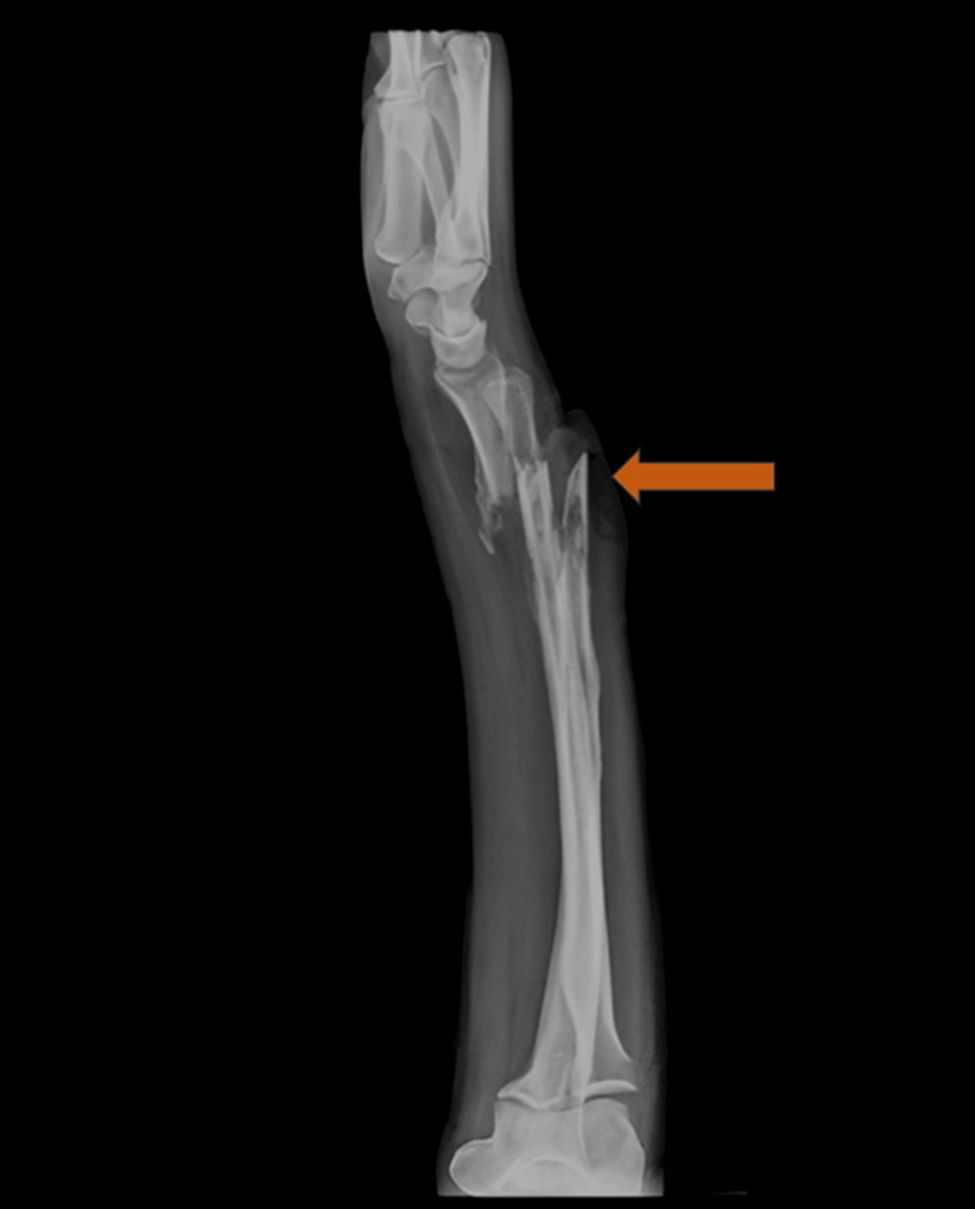
X-ray of the right forearm lateral view showing comminuted fracture with acute osteomyelitis of both bone distal forearm right (marked with orange arrow)

**Figure 8:**
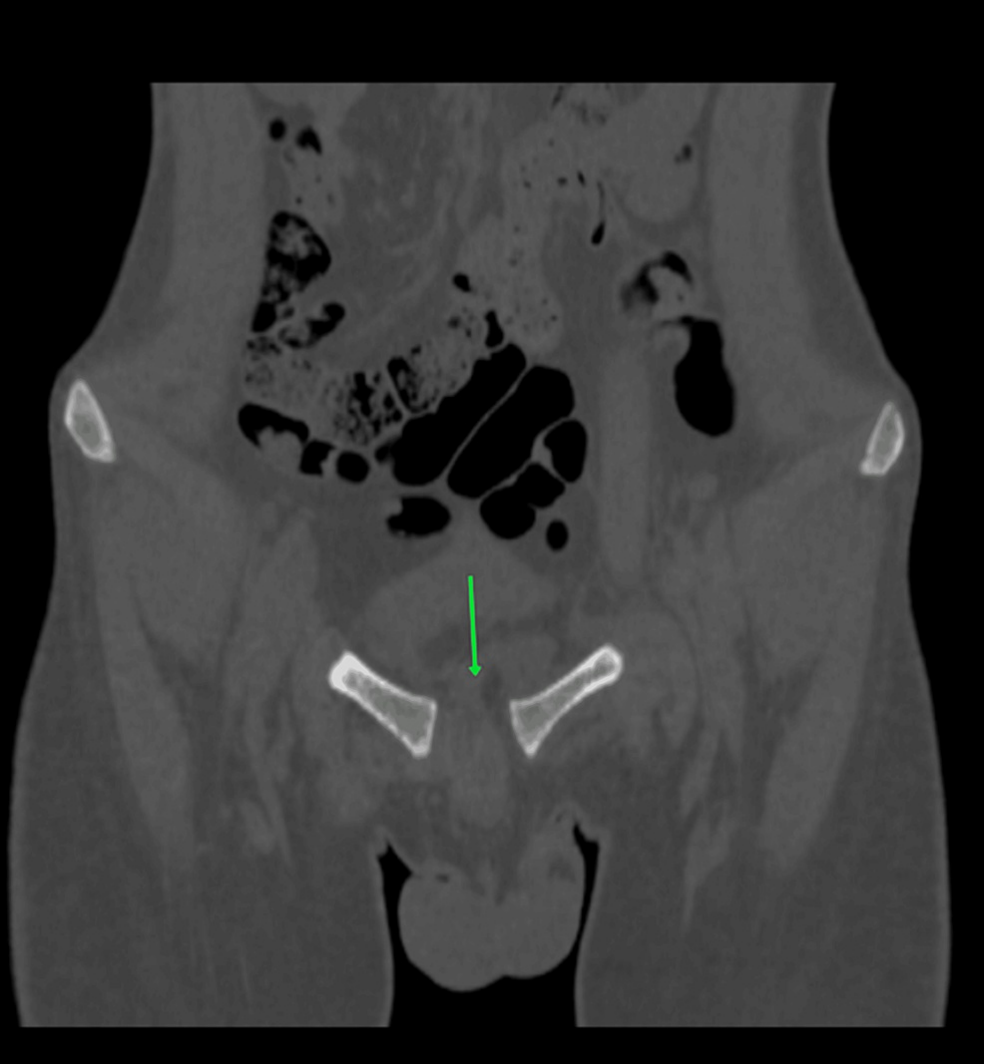
CT pelvis coronal cut view showing pubic symphysis diastasis (marked with green arrow)

**Figure 9:**
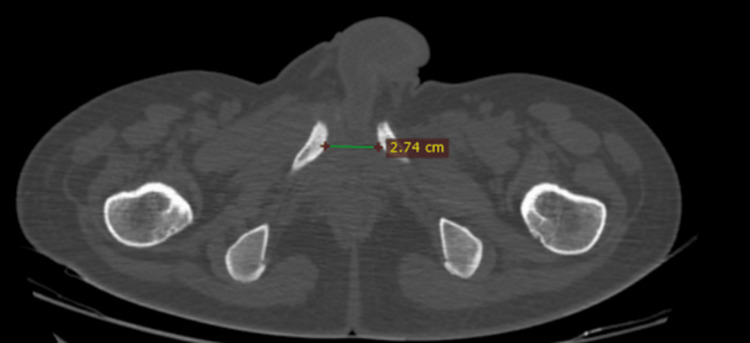
CT pelvis axial cut view showing pubic symphysis diastasis (2.74 cm)

**Figure 10:**
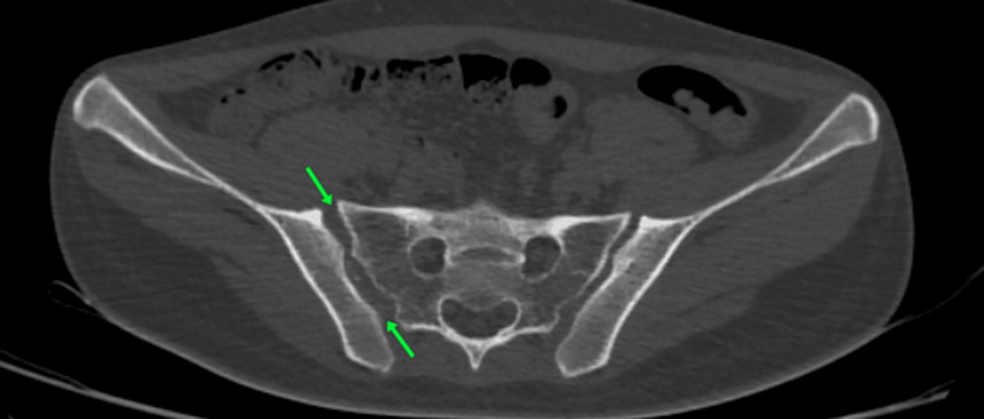
CT pelvis axial cut view showing right sacroiliac joint disruption (marked with green arrows)

**Figure 11:**
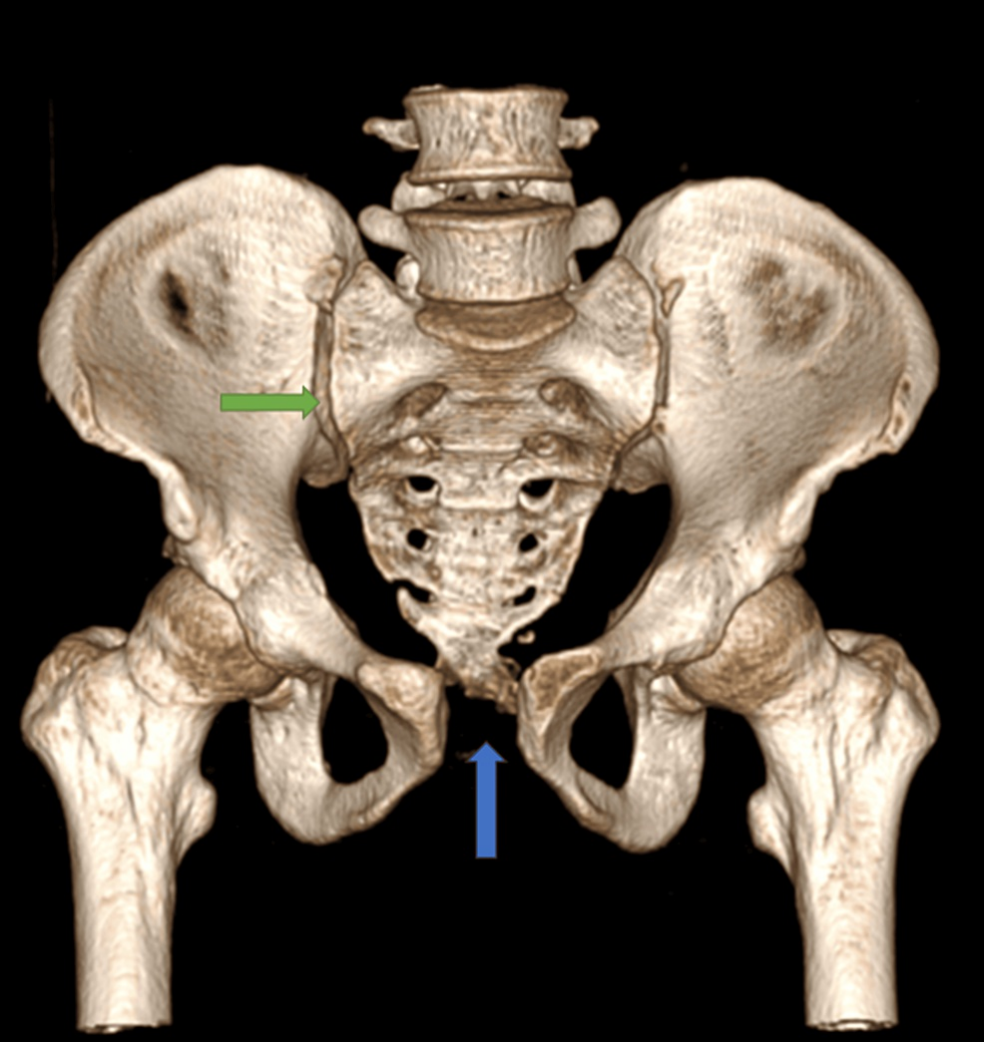
CT-3D anterior view of pubic symphysis diastasis (marked with blue arrow) and sacroiliac joint disruption right (marked with green arrow)

**Figure 12:**
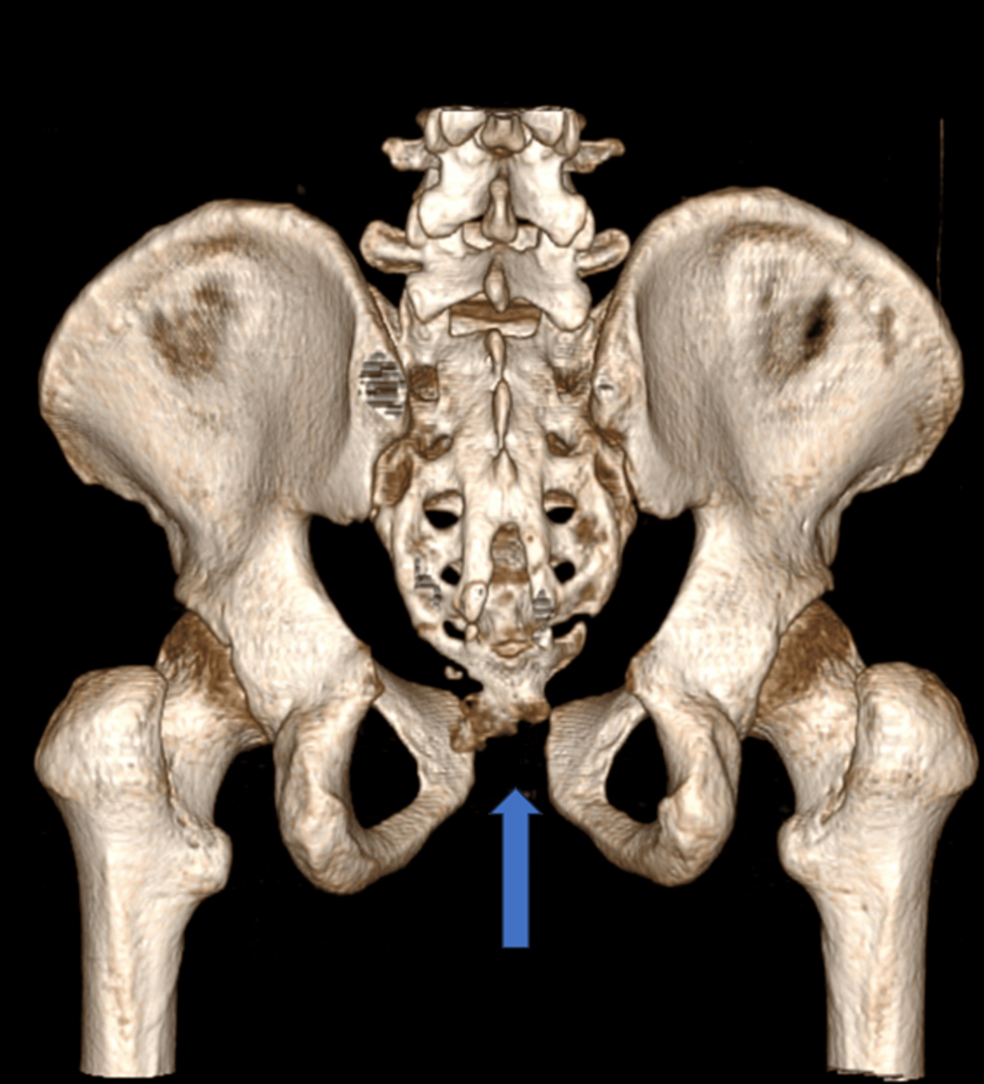
CT-3D posterior view of pubic symphysis diastasis (marked with blue arrow)

**Figure 13:**
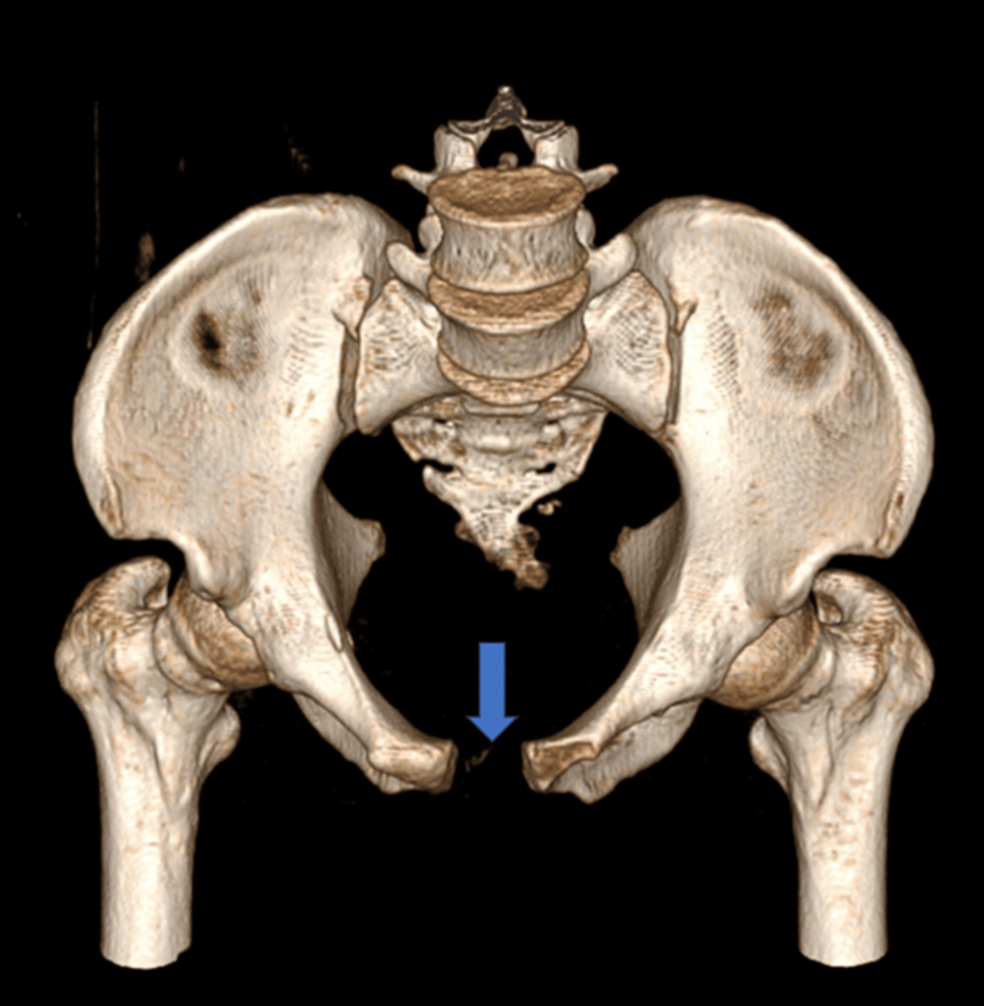
CT-3D inlet/superior view of pubic symphysis diastasis (marked with blue arrow)

**Figure 14:**
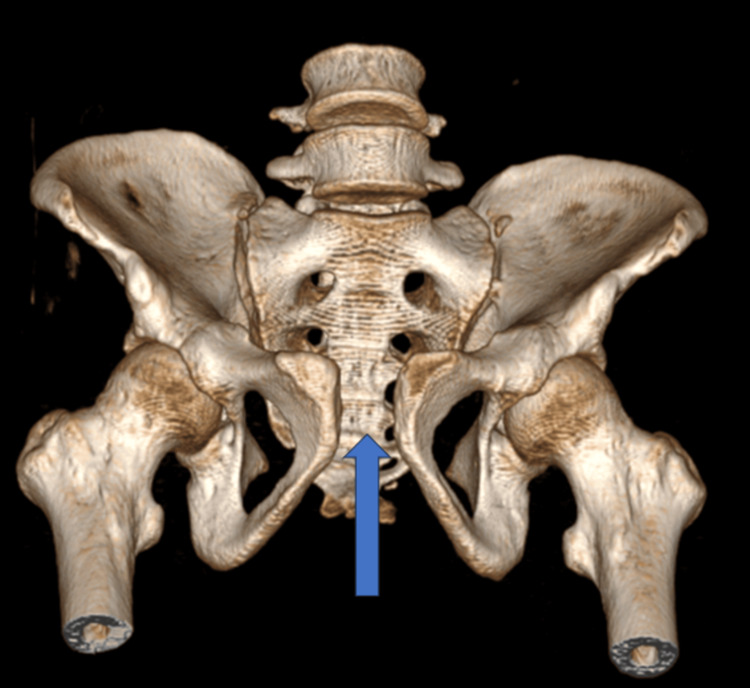
CT-3D outlet/inferior view of pubic symphysis diastasis (marked with blue arrow)

Preoperative radiographs of the pelvis (Figure 3), right thigh with the below-knee stump (Figures 4, 5), right forearm (Figures 6, 7), and preoperative CT pelvis (Figures 8-14) were performed. The pus from the right forearm wound site was sent for culture and sensitivity and was found to have the *Staphylococcus aureus* organism; the patient was started on sensitive antibiotics. The patient was diagnosed as a 2-month-old pubic symphysis diastasis and sacroiliac joint disruption right (Young and Burgess classification - APC-II, Tile classification - Tile B1), and 2-month-old comminuted fracture with acute osteomyelitis of both bone distal forearm right.

**Figure 15:**
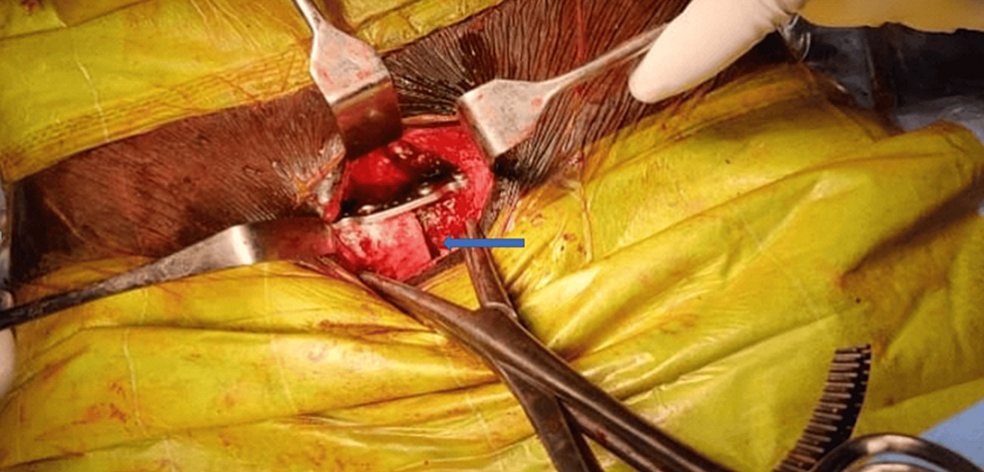
Intraoperative image showing pubic symphysis diastasis fixed with plating (marked with blue arrow)

**Figure 16:**
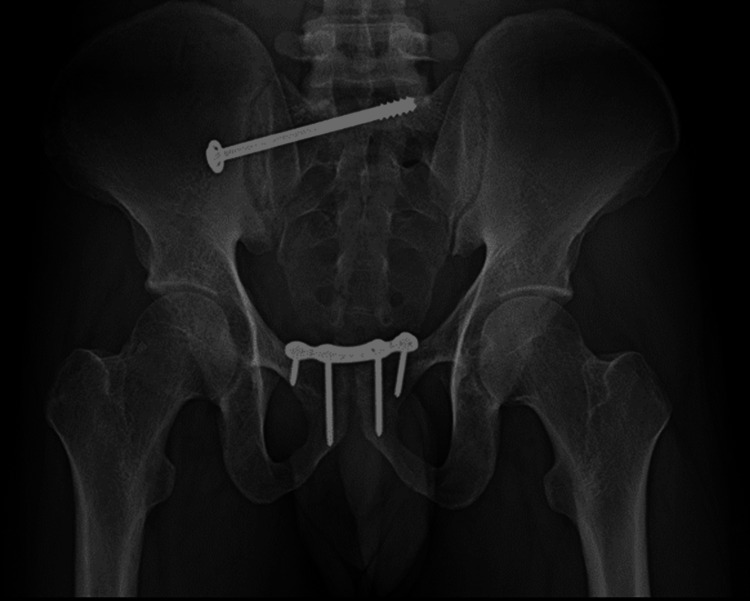
Postoperative X-ray of the pelvis outlet view

**Figure 17:**
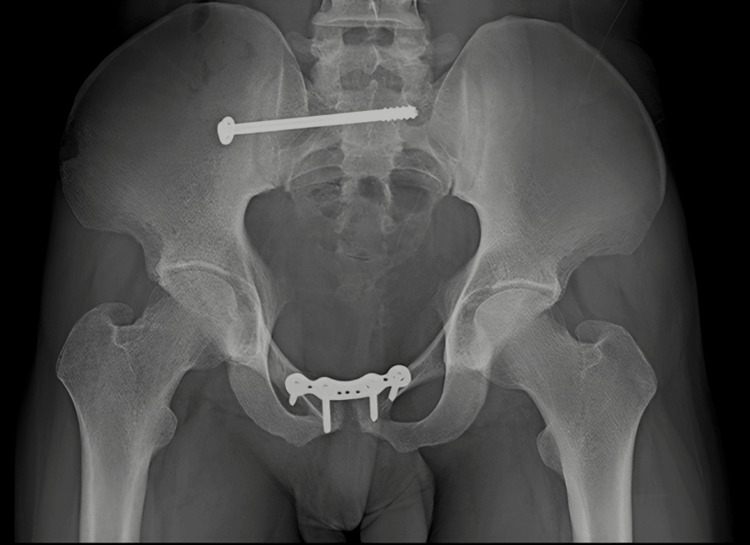
Postoperative X-ray of the pelvis inlet view

**Figure 18:**
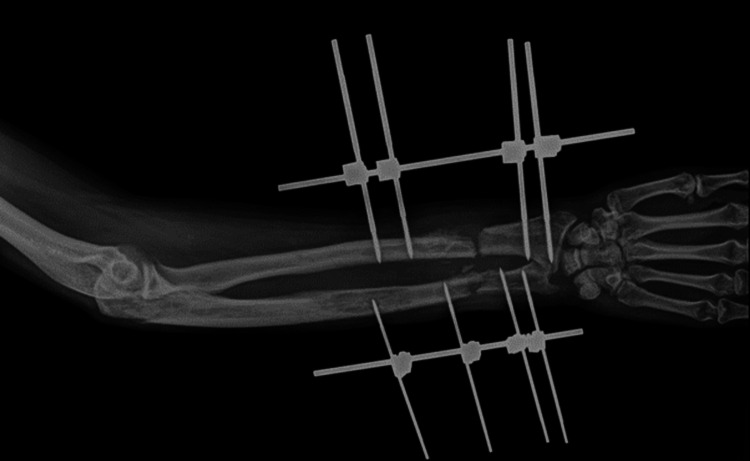
X-ray of the right forearm with the external fixator in situ

**Figure 19:**
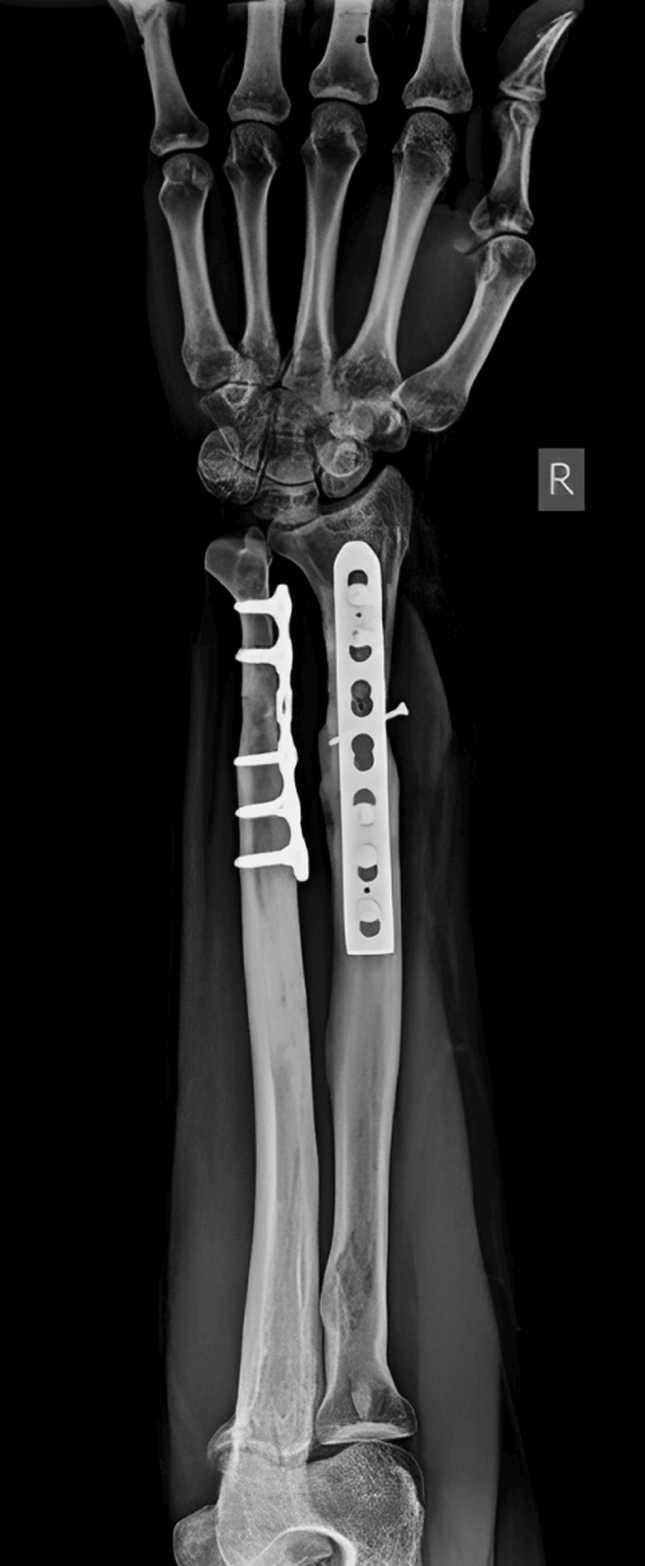
Postoperative X-ray of the right forearm anteroposterior view showing ORIF with plating of comminuted both bone fracture of the right forearm after removal of external fixator ORIF: open reduction and internal fixation

**Figure 20:**
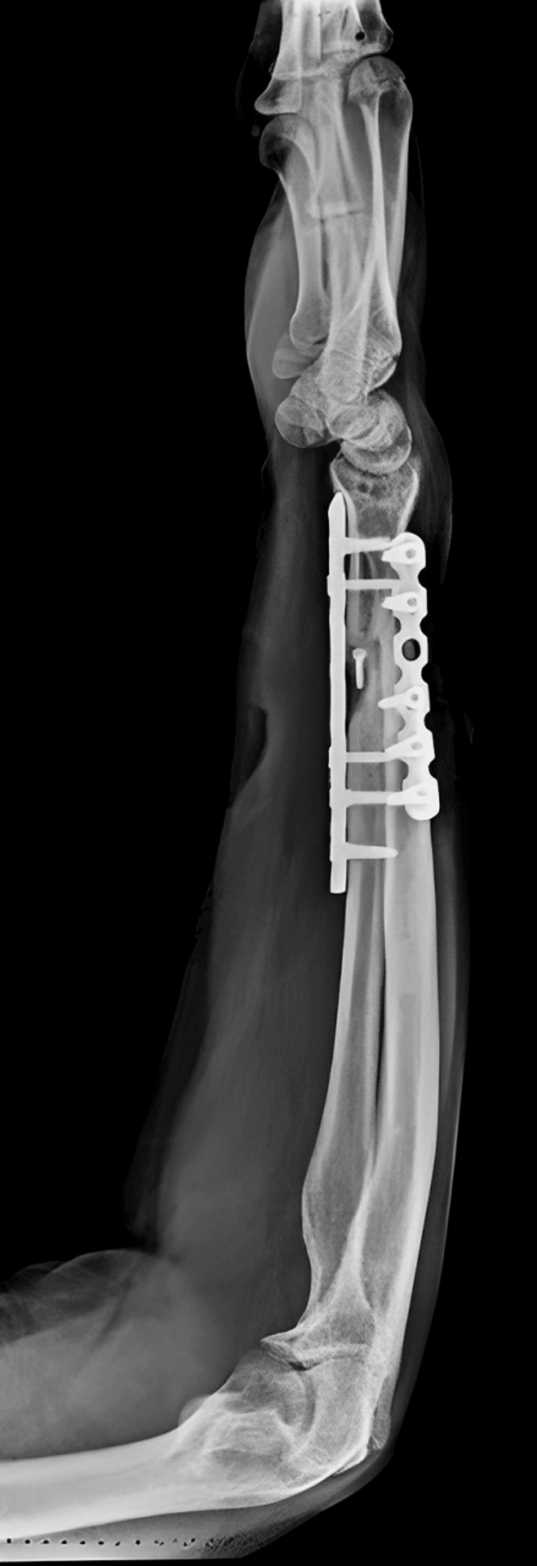
Postoperative X-ray of the right forearm lateral view showing ORIF with plating of comminuted both bone fracture of the right forearm after removal of external fixator ORIF: open reduction and internal fixation

The patient underwent surgical management open reduction and internal fixation with plating of the pubic symphysis diastasis and ilio-sacral screw fixation for right sacroiliac joint disruption (Figures 15-17) and wound debridement and external fixator application for 2-month-old comminuted fracture with acute osteomyelitis of both bone distal forearm right (Figure 18) and followed by open reduction and internal fixation with plating of comminuted fracture of both bone distal forearm right after resolution of acute osteomyelitis (Figures 19, 20) and sterile wound management is done for non-healing ulcer in right below knee stump. Postoperatively, the patient's hip range of movement improved, and erectile dysfunction resolved. 

## Discussion

Open book injury, characterized by a ruptured pubic symphysis and unilateral or bilateral opening of the sacroiliac joint, primarily occurs through two primary mechanisms: anteroposterior compression force and forced abduction and external rotation of one leg [1]. Pelvic fractures can result in significant long-term disability, including sexual dysfunction, bladder dysfunction, and bowel dysfunction [2]. Wright et al. reported an incidence of 21% and 8% for sexual and excretory dysfunction, respectively, among 298 pelvic fracture patients [7]. Male patients with sacroiliac joint injuries have a four-fold higher risk of developing sexual and excretory dysfunction [7]. Johnsen et al. investigated the anatomical factors associated with erectile function and found that 22.04% of patients with sacroiliac joint fractures and 33.17% of patients with pubic symphysis disjunction experienced erectile dysfunction [8]. Traction injuries to the presacral pudendal plexus and branches of the pudendal nerve may occur during sacroiliac joint disruption, resulting in unilateral and mild erectile dysfunction [9]. Compression and distraction in Tiles type B injuries and posterior ring disruptions in Tiles type C injuries can increase the risk of erectile dysfunction. Symphyseal diastasis (> 25 mm), sacroiliac fractures, lumbar transverse process fractures, and pubic ramus fractures are associated with a higher incidence of erectile dysfunction following pelvic fracture. In our case, the pelvic injury was initially neglected despite the implementation of initial life-saving measures. However, after surgical management of the neglected pelvic injury, the patient experienced significant recovery, including resolution of erectile dysfunction and improved hip range of motion.

## Conclusions

Neglected pelvic ring injuries are a potential source of pain, gait abnormalities, and urogenital symptoms. Prompt and judicious management of these injuries can help in faster recovery and reduce associated morbidity.
